# Caesarean section and post traumatic stress disorder

**DOI:** 10.1192/j.eurpsy.2023.2079

**Published:** 2023-07-19

**Authors:** H. Chebli, M. Chtibi, F. Azraf, F. Laboudi, A. Ouanass

**Affiliations:** University Psychiatric Hospital Arrazi in Sale, salé, Morocco

## Abstract

**Introduction:**

Childbirth can be a very painful experience, especially vaginally. It can have many complications. The obstetrical complications of caesarean section are well studied but its psychological complications are little mentioned. Most women recover quickly after giving birth, but others seem to have more difficulty. Researchers have attempted to identify perinatal risk factors for the development of post-traumatic stress disorder in parturients and caesarean section seems to be one of the predictors.

**Objectives:**

The objective of our study is to detect post traumatic stress disorder in women after vaginal delivery.

**Methods:**

This is a cross-sectional descriptive study conducted among women from the general population who have given birth vaginally. The information was collected using a questionnaire distributed on social networks. Symptom severity was quantified using the PTSD checklist for DSM-V (PCL-5).PCL-5 is a 20-item self-assessment that measures the 20 DSM-5 symptoms of PTSD. She rates each symptom from 0 (not at all) to 4 (extremely). The score varies from 0 to 80. A threshold of 33 allows screening for post-traumatic stress disorder.

**Results:**

61 women took part in this study, 81.5% of whom gave birth vaginally between the ages of 20 and 30.70.3% of the participants gave birth vaginally once, 44.4% twice and 7.3% of the women in our sample had a caesarean three times.Regarding the indication of the high way in our sample: the narrowing of the pelvis and fetal distress were in 22.2%. Exceeding term was in 18.5% of cases.In our sample, 59.3% of the women had planned their vaginal delivery and 40.7% had given birth urgently.Regarding the results of the PCL-5 scale (PTSD checklist for DSM-V), the score varies between 0 and 48 with a median score equal to 12. 3.7% of the participants had a score greater than 33.

**Conclusions:**

Post-traumatic stress disorder can accompany childbirth, especially when it is high and in an emergency. Postpartum post traumatic stress disorder affects the mother-child relationship and can be complicated by depressive disorder. Medical monitoring of pregnancy, good medical and family support for the parturient and preparation for childbirth are necessary to better start the maternity experience.

**Disclosure of Interest:**

None Declared

